# Association between hepatitis B virus infection and risk of osteoporosis: a systematic review and meta-analysis: A protocol for systematic review

**DOI:** 10.1097/MD.0000000000019719

**Published:** 2020-04-17

**Authors:** Xingwen Xie, Rui Huang, Xiuxia Li, Ning Li, Haijun Zhang, Shihong Xu, Dingpeng Li, Shanshan Xi, Kehu Yang

**Affiliations:** a Department of Orthopaedics, Affiliated Hospital of Northwest Minzu University; b Department of Orthopaedics, Gansu Second Provincial People's Hospital; c Clinical Medical College of Traditional Chinese Medicine, Gansu University of Chinese Medicine; d Evidence-Based Social Science Research Center, School of Public Health, Lanzhou University; e Key Laboratory of Evidence Based Medicine and Knowledge Translation of Gansu Province; f Department of Bone Oncology, Gansu Provincial Hospital of Tradition Chinese Medicine, Lanzhou, Gansu; g Department of Endocrinology and Nephrology, Hengshui Hospital of Traditional Chinese Medicine, Hengshui, Hebei; h Institute of Clinical Research and Evidence Based Medicine, Gansu Provincial People's Hospital; i Evidence-Based Medicine Center, School of Basic Medical Sciences, Lanzhou University, Lanzhou, Gansu, China.

**Keywords:** hepatitis B, meta-analysis, osteoporosis, systematic review

## Abstract

**Background:**

The potential association between hepatitis B virus (HBV) infection and development of osteoporosis has drawn significant attention from clinicians and researchers in recent years due to the increasing prevalence of HBV infection. This study aims to perform a systematic review and meta-analysis of the literature to show whether HBV infection is associated with an increased risk of osteoporosis.

**Methods:**

Case-control, cohort, and cross-sectional studies that report the incidence of osteoporosis, osteoporotic fracture, osteopenia, and bone mineral density level in populations with HBV infection will be selected. Four databases from their inception to October 2019 will be searched. All data were assessed and extracted by 2 authors independently. The Newcastle–Ottawa scale and (Agency for Healthcare Research and Quality) Agency for Healthcare Research and Quality checklist will be used to assess the quality of the selected studies. Stata 15.1 (Stata Corp, College Station, TX) will be used to conduct meta-analysis.

**Result:**

The results of this systemic review and meta-analysis will be submitted to a recognized journal for publication.

**Conclusion:**

This systemic review and meta-analysis will determine whether HBV infection is associated with an increased risk of osteoporosis. We hope this review can provide a reliable evidence.

**Registration:**

PROSPERO (registration number CRD42020140522).

## 1 Introduction

Osteoporosis is characterized by a reduction in bone mass without alterations in bone composition, which leads to higher tendency of fracture.^[1]^ It is estimated that there are as many as 200 million people worldwide with osteoporosis.^[2–4]^ In the United States, approximately half of all people over 50 years old are at risk for osteoporotic fracture.^[5]^ More than 60% of osteoporosis patients sustain an associated fracture in their lifetime and the high rate of disability and mortality associated with these fractures, especially in the year following a fracture, has increased the awareness of osteoporosis worldwide.^[6,7]^ Chronic liver disease can be associated with osteoporosis due to impairments in nutritional and biosynthetic processes and osteoporosis is a major complication in patients with chronic hepatitis.^[8,9]^

Epidemiological data suggests that the prevalence of osteoporosis is between 4% and 21% in patients with chronic liver disease and 10% to 43% in liver transplantation patients.^[10–12]^ High levels of tumor necrosis factor, lower levels of vitamin D, lower levels of insulin-like growth factor 1, and hypogonadism are considered to be possible mechanisms for bone loss in patients with chronic liver disease.^[13–16]^ This bone loss in patients with chronic liver disease is known as “hepatic osteodystrophy” and is a metabolic bone disease secondary to increased resorption and reduced formation of bone and hepatitis.^[17–20]^

Several recent studies have examined the potential association between hepatitis B virus (HBV) infection and measures of bone health, such as osteoporosis incidence or bone mineral density (BMD). However, not all of these studies have quantitatively analyzed these associations, and there have been no attempts to summarize the existing evidence in this area. Meta-analysis has been increasingly regarded as a high-quality method for determining research evidence to support treatment and clinical decisions.^[21–22]^ This study aimed to perform a systematic review and meta-analysis of the literature to determine if HBV infection is associated with an increased risk of osteoporosis.

## 2 Methods

### 2.1 Study registration and ethics

This protocol has been registered at PROSPERO (registration number: CRD42020140522; http://www.crd.york.ac.uk/PROSPERO). Ethical approval is not necessary for individual patients’ date will not be used in the systemic review and no privacy will be involved.

### 2.2 Selection criteria

#### 2.2.1 Type of study

We will include case-control studies, cohort studies, cross-sectional studies, the language of the literature will not be limited. Case reports, review articles, conference abstracts, editorials, letters, and expert opinions will be excluded.

#### 2.2.2 Participants

People in case group should be diagnosed as hepatitis B infection, no age, gender, race restricted. People in control group should be without hepatitis B infection.

#### 2.2.3 Exposure

The people regarded as exposure must be monoinfect HBV. In the same time they were without bone disease and other disease may cause osteoporosis. The control group will be healthy population that without hepatitis and other disease may cause osteoporosis.

#### 2.2.4 Outcomes

The outcomes will be explicitly reported as least one of the following: incidence of osteoporosis, incidence of osteoporotic fracture, incidence of osteopenia, or BMD.

### 2.3 Search strategy

A comprehensive online search will be conducted in 4 databases, including PubMed, Web of Science, Embase, and the Cochrane Database of Systematic Reviews, from their inception to October 1st 2019. The search terms “osteoporosis,” “osteopenia,” “bone mineral density,” “HBV,” “hepatitis B surface antigen,” and “hepatitis B virus.” The MeSH term “hepatitis” and “osteoporosis” will be used. The authors will also conduct a search of reference lists from relevant review articles and the final included studies for additional sources. For example, the search strategy for PubMed will be (((((((osteoporosis[Title/Abstract]) OR osteopenia[Title/Abstract]) OR bone mineral density[Title/Abstract]) OR BMD[Title/Abstract])) OR “Osteoporosis”[Mesh])) AND (((((hepatitis B virus[Title/Abstract]) OR hepatitis B suface antigen[Title/Abstract]) OR HBV[Title/Abstract])) OR “Hepatitis B”[Mesh]).

### 2.4 Data extraction

Data will be extracted by 2 authors independently. All differences in study inclusion/exclusion will be settled by discussion between the 2 authors. When there are differences between the 2 authors, a third independent reviewer will be consulted to make the final decision Data extracted includes the authors, journal, year of publication, study design, patient demographics, diagnostic criteria, and outcome measure (incidence of osteoporosis, incidence of osteopenia, incidence of fracture, or BMD).

### 2.5 Quality assessment

Assessment of the risk of bias of the included studies will be performed independently by 2 authors and was ranked as high, low, or uncertain. Possible discrepancies will be adjudicated by the other study-team members. All included studies will be cohort, case-control, or cross-sectional studies. For cohort and case-control studies, bias risk assessment will be conducted by using the Newcastle–Ottawa scale,^[23]^ which assesses the selection of study groups, comparability of study groups, and exposure measurement of a case-control study and the selection, comparability, and outcome measurement of a cohort study. Using that scale, 9 = maximum score, >6 is relatively high quality, 5 to 6 is fair quality, <5 is poor quality. The Agency for Healthcare Research and Quality checklist will be used to assess the risk of bias for the included cross-sectional studies.^[24]^ The Agency for Healthcare Research and Quality is composed of 11 items, with each item scored “0” if it will be answered “NO” or “UNCLEAR” and scored “1” if it will be answered “YES.” 11 = maximum score, ≥7 is relatively high quality, 4 to 6 is fair quality, ≤3 is poor quality.

### 2.6 Statistical analysis

#### 2.6.1 Meta-analysis

Meta-analysis will be performed by using Stata 15.1 (Stata Corp, College Station, TX). The outcome measures of interest for the meta-analysis will be osteoporosis incidence, osteopenia incidence, osteoporotic fracture incidence, and BMD. Pooled odds ratios with 95% confidence intervals will be calculated for dichotomous variables (disease incidence). Standardized mean differences with 95% confidence interval will be calculated for continuous variables (BMD).

#### 2.6.2 Measures for heterogeneity

Heterogeneity of the studies will be assessed by using the *I*^2^ index.^[25]^ If the *I*^2^ < 25%, studies will not be considered heterogeneous and a fixed effect model will be adopted for the meta-analysis; otherwise a random effects model will be adopted. If substantial heterogeneity detected, we will determine the cause of the heterogeneity by using subgroup and sensitivity analysis.

#### 2.6.3 Publication bias

Funnel plot and Egger test will be applied to evaluate the existence of publication bias.^[26]^

## 3 Discussion

Recently, several studies had paid attention to the association between HBV infection and risk of osteoporosis. The result was different. While few articles had summarized the existing evidence. As a result we cannot judge there association. Therefore, this systemic review and meta-analysis we conduct so as to provide reliable evidence. If they really have the association, in the future for patients with HBV infection. We should pay more attention to their bone health, because osteoporosis have a high rate of disability and mortality.

This protocol has been registered, we will strictly execute according to the Cochrane Handbook for Systematic Reviews of interventions and is presented per the preferred reporting items for systematic reviews and meta-analyses guideline.^[27,28]^

However, there may be several limitations in this systemic review. First, we only search 4 international database that may be lead to selection bias. While the 4 database have strong representative, maybe the representativeness is not affected. Second, we include different types of studies, for example case-control studies, cohort studies and cross-sectional studies, this may be cause substantial heterogeneity. Third, the diagnostic methods for HBV infection are varied, this may also be a source of heterogeneity. Fourth, this research is based on researches presently; the emergence of new researches in the future may have an impact on current results. We hope this systematic review and meta-analysis can provide a reliable evidence whether there is an association between HBV infection and the risk of osteoporosis.

## Author contributions

**Conceptualization:** Xingwen Xie, Rui Huang and Kehu Yang.

**Data curation:** Rui Huang and Xiuxia Li.

**Funding acquisition:** Xingwen Xie and Kehu Yang.

**Methodology:** Ning Li and Shihong Xu.

**Project administration:** Xingwen Xie.

**Protocol draft:** Xingwen Xie, Rui Huang and Xiuxia Li.

**Software:** Dingpeng Li, Shanshan Xi and Haijun Zhang.

**Study design:** Xingwen Xie and Kehu Yang.

**Validation:** Xingwen Xie and Kehu Yang.

**Writing – original draft:** Xingwen Xie Rui Huang.

**Writing – review & editing:** Xiuxia Li.
